# Human genetics, natriuretic peptides and hypertension

**DOI:** 10.1186/1471-2210-11-S1-O5

**Published:** 2011-08-01

**Authors:** Christopher Newton-Cheh

**Affiliations:** 1 Massachusetts General Hospital, Boston, MA, USA; 2 Broad Institute of Harvard and MIT, Cambridge, MA, USA

## Background

Hypertension (HTN) is a major worldwide cause of stroke, heart failure, myocardial infarction, and chronic kidney disease. Human genetics offers the potential to identify novel physiologic mechanisms that underlie blood pressure (BP). BP is a heritable trait but until recently genetic factors that influence BP at the population have been difficult to identify.

## Results

We used a candidate gene association study of common variants across the *NPPA*-*NPPB* locus to identify genetic variants that influence atrial natriuretic peptide (ANP) and B-type natriuretic peptide (BNP) [1]. The minor alleles of three noncoding SNPs rs5068 (MAF 0.06), rs198358 (MAF 0.19) and rs632793 (MAF 0.38) were associated with higher ANP (+0.42 SD p=8x10-70; +0.20 p=8x10-30; +0.08 p=2x10-10, respectively) and higher BNP (+0.17 SD p=3x10-12, +0.18 p=9x10-25, +0.21 p=2x10-68) in 14,515 individuals of European ancestry. The alleles of rs5068 and rs198358 associated with higher ANP/BNP were associated with lower SBP (p=2x10-6, p=6x10-5, respectively) and lower DBP (p=1x10-6, p=5x10-5) as well as lower odds of HTN (OR 0.85 p=4x10-5, OR 0.90 p=2x10-4) in 29,717 individuals. The association of rs5068 was replicated in the Global BPgen GWAS [2]. Recently, Kato et al reported the association of a common variant downstream of *NPR3* encoding the natriuretic peptide clearance receptor [3]. Murine NPR3 knockout is associated with lower BP, consistent with higher levels of circulating natriuretic peptides [4]. The minor allele of a SNP highly correlated to the BP *NPR3* SNP has recently been reported to be associated with taller stature in humans [5], consistent with the effect of murine knockout [4] or apparent loss-of-function mutations [6] in NPR3.

## Conclusion

The ANP-BNP/pGC/cGMP axis is important in the regulation of BP in humans. Further ongoing GWAS studies have identified additional novel loci. Human genetics can offer an entrée into the physiologic determinants of blood pressure at the population level and point to therapeutic opportunities to reduce the morbidity and mortality due to HTN.
